# The impact of employees' perceptions regarding hotels’ green intellectual capital on their environmental perceptions: A mediating moderation model

**DOI:** 10.1016/j.heliyon.2024.e39559

**Published:** 2024-10-18

**Authors:** Ertac Gulakdeniz, Georgiana Karadas

**Affiliations:** a Business Administration, Faculty of Economics and Administrative Sciences, Cyprus International University, Haspolat Campus, Northern Cyprus via Mersin 10, Turkey; b Department of Business Administration, Faculty of Economics and Administrative Sciences, Cyprus International University, Haspolat Campus, Northern Cyprus via Mersin 10, Turkey

**Keywords:** Green intellectual capital, Employees' thriving at work, Green hotels, Enterprise social media usage

## Abstract

Researchers acknowledge the significance of hotels' environmental performance, however they call for a further comprehensive examination of the aspects that serve as its drives. Moreover, despite the abundance of studies on environmental performance in the hospitality literature, employees' perceptions regarding hotels' green intellectual capital and its potential outcomes are scarcely investigated. With this realization, and building upon the job demand-resource theoretical framework and socially embedded theoretical framework of thriving, the current paper proposes a conceptual model where thriving at work mediated the impact of employees' perceptions regarding hotels' green intellectual capital on environmental performance moderated by work-related enterprise social media usage. To gauge these relationships, data was collected from frontline employees working in four and five star Turkish hotels. The study's interrelationships have been analyzed by employing structural equation modeling. The study revealed that employees' perceptions of hotels' green intellectual capital significantly influenced their thriving at work and positively shaped their views on hotels' environmental performance. Additionally, thriving at work demonstrated a significant impact on employees' perceptions of hotels' environmental performance, acting as a mediator between perceptions of green intellectual capital and environmental performance. Furthermore, the study found that the influence of employees' perceptions of hotels' green intellectual capital on thriving at work was moderated by work-related enterprise social media usage. According to our findings, top management should monitor and promote employees' perceptions of green intellectual capital, and their thriving at work, sustaining a green organizational culture at the same time via an effective usage of enterprise social media to foster environmentally friendly initiatives. This could involve creating platforms for idea-sharing and recognizing employees who implement sustainable practices.

## 1 Introduction

Due to the substantial growth occurring in the accommodation industry lately and the emerging trend of environmental sustainability (ES), hotel management is urged to show responsibility towards the environment by adopting eco-friendly activities [1]. Therefore, to preserve the environment, hotel managers are encouraged to activate their employees' eco-friendly behaviors, highlighting the hotels' willingness to invest in ES [2]. Although Khatter et al. [3] indicated that some hotels value commercial outcomes rather than environmentally sustainable practices, the hospitality industry has shown a great interest in ES and has capitalized on green management to enhance hotels’ environmental performance (EP) [4]. For example, Hilton Worldwide and Marriott International are two of the largest hotel chains that have developed and implemented eco-friendly programs to preserve the environment [5].

Hotel environmental performance (EP) refers to the hotels' green efforts, comprising waste reduction, water and energy conservation in operations, as well as, employees' and customers' educational practices [1; [6]; 7]. It is quite known that hotels are responsible for consuming the largest amount of resources, primarily for purposes such as heating, cleaning, and lighting, resulting in the generation of tons of waste [8]. Accordingly, human resource management (HRM) in hotels adopts green management practices and focuses on enhancing employees' perceptions regarding hotels’ green intellectual capital (GIC) to contribute to their EP [9].

GIC comprises both intellectual capital and environmental endeavors [10]. It refers to the knowledge, capabilities, and experience of environmental initiatives (EI) [1]. It is known that frontline employees, who have the needed skills, abilities, and knowledge, would get engaged in environmental activities [11; 12]. Spreitzer and Hwang [13] describe thriving at work (TAW) as a psychological condition characterized by the feelings of vitality and learning that one may encounter in their work environment. Learning refers to the individual's tendency to grow and develop through acquiring and implementing further knowledge and skills, whereas vitality represents the propensity of an individual to feel energized in a given workplace [14]. Therefore, it is expected that when employees' GIC perceptions are high, their (TAW) and their perceptions of green EP will be directly and indirectly affected; that is, TAW acts as an underlying mechanism in the above-mentioned relationship. Meanwhile, in the era of technology and innovation, organizations tend to rely on enterprise social media (ESM) to enhance coordination, collaboration, and interpersonal communication among workers [15]. Work-related ESM is a web-based platform used to share information among co-workers within an organization [16]. Accordingly, employees can read and exchange work-related information and share advice through ESM thus demonstrating higher commitment [17], TAW [18], resource generation, and team collaboration [19].

According to Job Demands-Resources (JD-R), employees get engaged in their work when they have access to job resources (JR) [20; 21]. In a recent empirical investigation by Jiakui et al. [22], GIC was examined as an essential JR and its valuable outcomes were investigated. Moreover, Bakker and Demerouti [20] argued that workers who are energized and have the tendency to learn and grow, would demonstrate better performance. Accordingly, these employees would contribute to their organizations via their environmentally sustainable activities [23]. The socially embedded model (SEM) of thriving by Spreitzer et al. [24] is a crucial theoretical framework used to study the causes and important results of TAW, as discussed by Chang and Busser [25] and Zhu et al. [18]. Porath et al. [14] noted that there are several factors that would affect TAW, such as agentic work behaviors, JR, and work contextual features. Meanwhile, in line with the SEM of thriving, Bodhi et al. [26] and Zhu et al. [18] revealed that work-related ESM usage enhances information and knowledge sharing in a given workplace, thus triggering employees’ thriving.

The present study presents numerous contributions to the literature in hospitality and tourism industry. Initially, it is well known that hotels, which are engaged in eco-friendly initiatives, need employees who consistently provide exceptional service to attain and maintain customer satisfaction, especially green satisfaction [27; [28]; 29]. Broadly speaking, ES, representing a new shift in our current era, highly depends on employees', management's, and other stakeholders' commitment to green behavior [1]. However, previous empirical studies have investigated consumers' attitudes and perceptions towards EP without taking into consideration employees' behavior and perceptions [30; 31]. Hence, this paper examines the antecedents of employees' perceptions regarding a hotel's EP. Moreover, various empirical studies have advocated for additional investigation to determine the effect of employees' perceptions regarding GIC in service industries [32]. Evaluating employees' perspectives on GIC in the hospitality industry is crucial because of its significant resource consumption and waste generation [33]. Accordingly, hotels would adopt various greening activities to accomplish ES [32]. With this realization, it is of utmost importance to examine the effect of employees' perceptions regarding GIC on hotels' EP.

Second, the literature review delineates a few empirical studies linking GIC to TAW and EP. For instance, Malik et al. [11] claimed that investigations regarding GIC are in their early phases and called for further research. Similarly, other researchers have called for further investigations to determine the consequences of GIC as a means to widen the scope of green HRM and promote sustainable outcomes [34]. In particular, few papers have examined employees’ perceptions regarding GIC and their impact on their perceptions of EP [9]. Meanwhile, other researchers inferred that GIC is a novel variable that warrants further investigation in service industries [1]. Therefore, the present paper will provide further insights into the environmentally sustainable literature.

Third, employees, who thrive at work develop positive feelings and become more energized in their workplace, demonstrating better work performance [35] and displaying environmental behavior (EB) [33]. Consistent with the JD-R theory, thriving employees would have a higher tendency to learn and grow [20]. Accordingly, these employees would demonstrate EB's in the presence of essential JR such as GIC [22]. However, previous empirical studies suggested further research to investigate the antecedents of TAW and its valuable outcomes [36]. Therefore, ascertaining whether TAW influences employees' perceptions regarding hotels' EP is imperative. Moreover, to our knowledge, no empirical study has investigated the effect of employees' perceptions regarding hotels' GIC on EP via TAW. In this sense, our paper tends to assess the mediating role of TAW in the aforementioned linkage.

Fourth, this paper utilized the SEM of thriving and the JD-R model, linking employees' perceptions regarding GIC to EP via TAW. As propounded by the SEM [24], Jang et al. [37], as well as Porath et al. [36], revealed that employees’ thriving is highly affected by the work contextual features such as a supportive work environment, organizational culture, trust, knowledge sharing, and decision-making discretion, as well as by the resources such as relational resources and high-quality relationships.

Finally, employees can share information and interact socially via work-related ESM within organizations. ESM supports internal communication and collaboration among workers, influencing various work outcomes [26]. Although previous papers have investigated human-computer interaction focusing on special technologies [16], scarce studies have investigated the implications of employees' perceptions regarding work-related ESM usage [15; 38] With this recognition, our study examines employees' perceptions regarding work-related ESM usage as a moderator between employees' perceptions of hotels’ GIC and EP via TAW.

Drawing on past empirical research and guided by the principles of the JD-R theory and SEM of thriving, the paper aims to: a) explore how employees' views on GIC influence their TAW and their perceptions of hotels' EP b) investigate the impact of TAW on employees' views of hotels' EP c) evaluate the intermediary role of thriving between employees' perceptions of hotels' GIC and EP, and d) examine how employees' perceptions of work-related environmental social media usage moderate the aforementioned connections.

In summary, this research will provide empirical evidence regarding employees' perceptions of hotels' GIC, their positive influence on TAW, and their perceptions of the hotels' EP.

Moreover, TAW may act as a mediator between employees' perceptions of GIC and EP. That is, employees' perceptions of GIC influence their levels of TAW, which consequently can determine their perceptions regarding the hotels’ EP.

The study will also reveal the moderating role of employees' perceptions of work-related ESM on the relationship between GIC perceptions and TAW; that is, the higher the employees’ perceptions of ESM are, the stronger the above-mentioned relationship will be.

The following part of the paper covers the background, development of hypotheses, technique description, and data collection from frontline personnel in four- and five-star Turkish hotels. The results and their interpretations are also provided. The paper's final section presents the conclusion, along with the theoretical and managerial implications, limitations, and future recommendations of the research.

## 2 Literature review

### 2.1 Theoretical background

The JD-R model, according to Bakker and Demerouti [20], is a theoretical framework that explores the dynamics between job demands and JR and their effects on employee outcomes [20]. At its essence, the model proposes that each job encompasses distinct demands and resources. Job demands are the aspects of a job, whether physical, psychological, social, or organizational, that require continuous effort and are linked with psychological and physiological costs [39]. On the other hand, JR refers to aspects of a job that support employees in achieving their work objectives, lessen job demands, and promote personal growth, learning, and development [40]. The JD-R model outlines that the interaction between job demands and JR triggers two key psychological processes: the health impairment process and the motivational process. The health impairment process arises when job demands surpass what an employee can manage, resulting in stress, burnout, and reduced well-being [20]. In contrast, the motivational process is initiated when JR is plentiful, enhancing motivation, engagement, and overall well-being. Conversely, the motivational process occurs when JR are abundant, leading to increased motivation, engagement, and well-being. Previous investigations [1; [41,42]; 43] utilized the JD-R model to explain the relationship between GIC and EP.

Several studies [1; [41,42]; 43] have also investigated the mediating role of ambidextrous green innovation, green HRM, EB, and environmental responsibility between GIC and EP, using the JD-R model.

In addition, the SEM of the thriving was employed to demonstrate how TAW mediates the relationship between organizational inducements and work outcomes [44]. Specifically, Karadas et al. [44] highlight that under the SEM of thriving, employees' views on JR within hotels, like GIC, play a significant role in fostering vitality, personal development, and learning in the workplace. Furthermore, these perceptions about hotel JR not only contribute to employee’ TAW but also shape their views on hotels’ EP [1; 42].

### 2.2 Hypotheses development

#### 2.2.1 Green intellectual capital, environmental performance, and thriving at work

GIC comprises intellectual capital and EI [1; 10] Intellectual capital is a concrete resource referring to individuals' experience, knowledge, and abilities that would improve their performance, thus enhancing organizations' competitive advantage [45]. Meanwhile, individuals’ GIC refers to the skills, knowledge, and capabilities of environmental greening, thus allowing the transformation of environmental goals into pro-environmental actions [12].

According to Chen [10], GIC is a three-dimensional construct. The first dimension of GIC is the human capital, which comprises the overall capabilities, knowledge, and skills needed to achieve and maintain organizational goals. The second dimension is structural capital, known as organizational capital, and it refers to organizational assets such as organizational culture, capabilities, databases, hardware, software, and trademarks. The structural capital is considered to be the supportive basis for the human capital. The third dimension is relational capital which refers to the loyalty, trust, and goodwill of customers towards the organization, partners, channels, suppliers, and other stakeholders.

Previous empirical studies explored the consequences of employees' perceptions regarding GIC. For example, Yong et al. [32] inferred that green HR practices would significantly influence employees' perceptions of GIC and organizational environmental sustainability. Malik et al. [11] revealed that employees' positive perceptions regarding human capital enhance organizations' EP and motivate them to reduce waste production. Moreover, Singh et al. [12], as well as Yusliza et al. [34], indicated that GIC is an essential JR to enhance employees' behavior towards environmental greening, thus improving organizations' EP. Recently, Nisar et al. [1] revealed that employees' perceptions regarding GIC have a significant impact on their perceptions' regarding hotels' EP via EB. In the hospitality context, EP refers to the environmental activities being implemented by hotels' management to minimize their negative implications for the environment [46]. There is a significant gap in research on how employees' perceptions of GIC affect their perceptions of hotels' EP. It is obvious that an organization's success in environmental management highly relies on its employees' eco-friendly behavior. Consistent with the JD-R theoretical framework, GIC is considered an essential JR that would influence employees' work outcomes [22]. Accordingly, employees who realize that various JR are in place would demonstrate eco-friendly behaviors [21]. Indeed, employees' eco-friendly behaviors would positively influence organizations' EP [5].

Meanwhile, TAW has received increasing attention among scholars and practitioners [25; 47]. For instance, Chénard-Poirier et al. [48], as well as Rahaman et al. [49], revealed that contextual factors such as job autonomy and workplace support, as well as, individual factors such as personality, highly affect thriving. Moreover, Jiang et al. [50] indicated that TAW empowers employees, thus helping them to develop an effective direction and perform their work efficiently. Additionally, Kim et al. [47] noted that TAW would enhance job performance and promote heedful relationships. According to Jiang [55], the impact of personal factors such as core self-evaluation and proactive personality and personal barriers such as negative affect and identity conflict [56] influenced TAW, and a deeper understanding of GIC's influence on thriving is significantly needed.

It is known that thriving targets the cognitive and affective basis of personal growth [51]. Specifically speaking, learning (cognitive) represents employees' ability to acquire and implement further knowledge leading to personal growth and development at work; whereas, vitality (affective) refers to the feeling of owning energy at work [24]. Ultimately, individuals, who lack one of these dimensions, cannot thrive [51]. As postulated by the SEM of thriving [24], vitality and learning are deeply incorporated within individuals' social systems, and the available resources existing in the work environment, such as motivational, informational, emotional, and relational resource, promote thriving through individuals' agentic work behaviors [13; 52]. Recent empirical studies revealed that employees' perceptions regarding the relational resources among leaders, co-workers, and customers highly influence employees' TAW [47; 53]. Meanwhile, researchers indicated that learning and vitality enhance sustainable outcomes that would yield positive outcomes [51]. As advocated by the JD-R model, employees who exhibit positive feelings and are energetic, would become more motivated and get more engaged in their work [20]. Therefore, workers would demonstrate positive outcomes contributing to their organization's EP via their eco-friendly behaviors [4; [23]. It is quite known that employees' environmental knowledge significantly influences their EP [1]. Specifically, environmental skills, knowledge, and abilities would influence employees' perceptions regarding their organization's EP [32]. Moreover, Basinksa and Rozkwitalska [54] claimed that TAW helps individuals achieve self-development and valuable work goals. In line with the SEM, job-related resources would predict TAW [50; 57].

As posited by the JD-R theory, SEM of thriving, and the aforementioned results, we contend that employees' perceptions regarding hotels' GIC would influence personal growth, and learning at a given workplace. In addition to exhibiting thriving, employees' perceptions regarding hotels' GIC would influence their perceptions of hotels’ EP. Hence, we postulate that.

Hypothesis 1 (H1): Employees' perceptions regarding GIC have a positive impact on a) their perceptions toward hotels' EP, b) learning, and c) vitality.

Hypothesis 2 (H2): a) Vitality and b) learning have a positive impact on employees' perceptions regarding hotels' EP.

#### 2.2.2 The mediating role of thriving at work

Various empirical studies have investigated the outcomes of TAW. For instance, TAW highly influences heedful relating [47], reduces employees’ negative attitudes, perceptions, and behaviors in a given workplace [25; 58], and promotes positive work outcomes [47].

The JD-R theory argues, GIC can be a JR [10; 22], stimulating employees’ development, learning, and growth. Moreover, as posited by the SEM of thriving, vitality and learning are deeply incorporated within an employee social system and are highly impacted by the resources existing in the work context [36]. According to Spreitzer and Hwang [13], as well as Rego et al. [52], JR such as relational, informational, emotional, and motivational resources are developed while doing work and would predict TAW.

In congruence with the JD-R theory, SEM, and previous empirical studies, we present a theoretical approach that investigates how employees' perceptions of GIC affect EP through TAW. Hence, we posit.

Hypothesis 3a (H3a): Vitality has a mediating role between employees' perceptions regarding hotels' GIC and hotels' EP.

Hypothesis 3b (H3b): Learning has a mediating role between employees' perceptions regarding GIC and hotels' EP.

#### 2.2.3 The moderating role of work-related ESM usage

Most organizations, nowadays, adopt various social networking applications to promote socialization, foster socio-emotional relationships, nurture trust development, and improve organizational performance [59]. Work-related ESM usage promotes communication visibility and induces employees' interactivity, cooperation, team collaboration, and coordination [19]. It supports knowledge sharing, communication, and interaction among employees within an organization, thus nurturing employees’ sense of belonging [38].

ESM plays a critical role in triggering trust among employees in online communities [60], increasing peer awareness and building relationships based on mutual reliance [38], generating knowledge and enhancing the flow of worthy information and valuable resources [26], promoting collective intelligence, team collaboration, and sharing of information [19], increasing employee’ learning abilities in different domains and energizing them in a given workplace [61; 62], allowing strategic usage of information, as well as enhancing employees' knowledge-based collaborations, enhancing employees’ commitment, increasing work efficiency [63], and improving management, decision-making and cross-boundary interactions [15].

Within the hospitality industry, knowledge exists in the form of internal and external relationships, system and business processes, and databases [1]. Meanwhile, GIC exists in the form of skills, knowledge, commitment, and creativity, thus helping employees in hotels reduce waste production and perform sustainably [11]. As posited by the JD-R theory, workers who are equipped with the needed resources, skills, and knowledge will demonstrate eco-friendly behaviors. Specifically speaking, human, structural, and relational capital are intangible assets highly correlated with EP [4]. Therefore, ESM usage within organizations would play an essential role in transferring knowledge, skills, and information regarding EI among employees.

It is quite known that work-related ESM usage enhances internal communication, allowing employees to acquire, transfer, and share information within an organization [26]. Accordingly, workers would exhibit personal development and vitality, thus triggering TAW [18; [61,62]; 64] enhancing employees' knowledge regarding unexpected environmental changes [65], nurturing organizations’ sustainable outcomes [50; 51], and enhancing their perceptions of EP [4; 5]. Tang et al. [66] determined that using ESM in a workplace is considered wasteful due to the inefficient use of online resources, it does not align with organizational functions and needs [67]. Moreover, according to Zhang et al. [59], researchers claimed that there is a scarcity of empirical evidence concerning the advantages and disadvantages of ESM usage concerning work performance. Considering the information provided, we advance that.

Hypothesis 4 (H4): The relationship between employees' perceptions regarding hotels' GIC and a) learning and b) vitality will be stronger when employees demonstrate positive perceptions regarding work-related EMS usage.

**Fig. 1:**
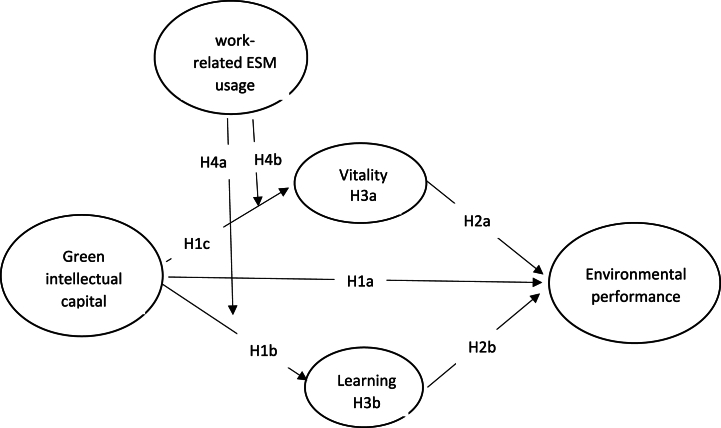
Research model.

Fig. 1 demonstrates the conceptual framework of the investigation.

## 3 Methodology

The study's deductive approach is grounded in a structured and systematic framework, emphasizing the testing of specific hypotheses derived from existing theories or established principles [68]. This methodological choice reflects a deliberate decision to follow a logical sequence, starting from general principles and theories and narrowing down to specific observations and conclusions [68]. The deductive approach is inherently aligned with the study's objective of generating empirical evidence that contributes to the advancement of knowledge in a particular field. By adopting a deductive approach, the researchers aim to hypothesize certain relationships and draw conclusions based on the data collected.

Data was collected by administering a carefully crafted questionnaire. This survey instrument was designed to gather relevant information from the study participants in a structured manner, aligning with the deductive nature of the research.

Data was collected from frontline employees employed in hotels situated in the three most frequented tourist provinces of Turkey.

Convenience sampling was employed in this study to gather data from frontline hotel employees across three provinces [42; 69]. The convenience sampling could be usually implemented as it denotes ‘‘the collection of information from members of the population who are conveniently available to provide it’’ [70], therefore, it can be easily and quickly applied until the required sample is reached [71]. In addition, in line with previous studies which examined thriving at work such as [42; [47]; 72] the present study considered it appropriately to implemented it.

The research selected the hospitality industry for several reasons. First, the researchers targeted four- and five-star hotels located in highly visited places such as Istanbul, Antalya, and Muğla (Turkish Ministry of Culture and Tourism, 2022). Second, customer-employee interaction highly influences the service delivery process and plays a significant role in delivering value to customers as a means to achieve customer attainment and loyalty [73]. Third, frontline employees play an important role in helping the hotel management achieve environmentally sustainable goals [74].

The hotels, that had been contacted, were selected from the website of the Directorate of Certification and Development of Tourism Enterprises department within the Ministry of Culture and Tourism of the Republic of Turkey. In particular, the participating hotels were selected from the 274 hotels classified as four- and five-star environmentally friendly hotels.

The process of gathering data was overseen by the managers of the hotels. Data were collected between May and July 2022 from employees via an online survey constructed on the Google Form platform and distributed via the organization's ESM. Three hundred and eighty questionnaires were sent to the participants. Three hundred and seventy-three questionnaires were returned. Two questionnaires were discarded since they showed incomplete information. Therefore, 371 responses were utilized for analyzing the data. The rate of response was 97.6 %, which is considered an acceptable result in social science studies, according to Hair et al. [75].

To reduce common method bias, this study adopted various procedural safeguards suggested by Podsakoff et al. [76]. Furthermore, the study emphasized the voluntary nature of participation, ensuring individuals felt no obligation to partake. It was also clearly communicated to the participants that the survey questions did not have correct or incorrect responses, reinforcing the importance of honest and spontaneous answers.

### 3.1 Measurement items

The measures for the variables in this study were derived from established scales in prior empirical research. The survey was originally created in English, then translated into Turkish, and finally back-translated into English to ensure accuracy. The Cyprus International University Scientific Research and Publication Ethics Committee sanctioned the questionnaire on April 4, 2022. A preliminary test with 20 frontline employees indicated that the survey's wording was clear and required no modifications. Participants provided responses to all survey questions using a 7-point Likert scale, with options ranging from 1 (strongly disagree) to 7 (strongly agree).

The construct of GIC was defined according to the framework set out by Chen [10], utilizing three dimensions: five items of measured green human capital, eight items of green structure capital, and five items of green relational capital. The concept of TAW was measured using 10 items based on Porath et al. [14], encompassing two aspects: learning and vitality. Kim et al. [5] provided the basis for measuring EP with seven items, and the measurement of work-related ESM usage was based on five items from Chen and Wei's [15] research.

### 3.2 Statistical technique

The respondents' demographic characteristics were thoroughly examined using the Statistical Package for the Social Sciences (SPSS) version 25, employing descriptive statistics analysis.

Table 1 summarizes the demographic characteristics of the participants. Most participants (223, 60.1 %) were female, while 144 (38.8 %) were male. A significant portion of respondents (185, 49.9 %) fell within the age range of 25–34 years, with 142 (38.3 %) falling within the 35–44 year age bracket. Additionally, 28 (7.5 %) respondents were younger than 25 years old, and 15 (4 %) were aged between 45 and 54 years. In terms of educational attainment, 274 (73.9 %) respondents had completed a 4-year college degree, 35 (9.4 %) had completed a 2-year college degree, and 37 (10 %) were postgraduates. Regarding tenure in their current position, approximately 29 % (108) had tenures between 3 and 5 years, 27.8 % (103) had tenures between 5 and 10 years, and 23.2 % (86) had tenures between 1 and 3 years. Meanwhile, 7.5 % (28) had tenures of less than 1 year, and 12.4 % (46) had tenures of more than 10 years (see Table 2).

**Table 1:** Participants’ demographic profile.

Demographic variables	Categories	Frequency	Percentage
Gender	Male	144	38.8 %
	Female	223	60.1 %
	Prefer not to Say	4	1.1 %
Age	<25	28	7.5 %
	25–34	185	49.9 %
	35–44	142	38.3 %
	45–54	15	4.0 %
	>25	1	0.3 %
Educational level	High school graduate and below	25	6.7 %
	2-year college	35	9.4 %
	4-year college	274	73.9 %
	Postgraduate	37	10 %
Tenure	<1 year	28	7.5 %
	1–3 years	86	23.2 %
	3–5 years	108	29.1 %
	6–10 years	103	27.8 %
	>10 years	46	12.4 %

**Table 2:** Validity and reliability assessment.

Construct and measurement items	Outer loadings	AVE	CR	Cronbach's alpha	Rho-A
Career intellectual capital		0.520	0.951	0945	0.948
GHCQ1	0.815				
GHCQ2	0.815				
GHCQ3	0.736				
GHCQ4	0.647				
GHCQ5	0.689				
GSCQ6	0.642				
GSCQ7	0.699				
GSCQ8	0.638				
GSCQ9	0.699				
GSCQ10	0.709				
GSCQ11	0.649				
GSCQ12	0.701				
GSCQ13	0.725				
GRCQ14	0.728				
GRCQ15	0.800				
GRCQ16	0.752				
GRCQ17	0.784				
GRCQ18	0.708				

Learning		0.762	0.941	0.921	0.929

LearningQ1	0.895				
LearningQ2	0.909				
LearningQ3	0.909				
LearningQ4	0.776				
LearningQ5	0.869				

Vitality		0.743	0.920	0.884	0.885

VitalityQ1	0.852				
VitalityQ2	0.881				
VitalityQ3	0881				
VitalityQ4	0.832				

Work-related ESM usage		0.760	0.840	0.920	0.927

ESM Q1	0.893				
ESM Q2	0893				
ESM Q3	0.893				
ESM Q4	0.897				
ESM Q5	0.776				

Hospital environmental Performance		0.659	0.931	0.913	0.9147

HEPQ1	0.848				
HEPQ2	0.837				
HEPQ3	0.840				
HEPQ4	0.777				
HEPQ5	0.780				
HEPQ6	0.834				
HEPQ7	0.761				

Note. GHC: green human capital; GSC: green structure capital; GRC: green relation capital; ESM: Enterprise social media; HEP: Hospital environmental performance.

SmartPLS is software designed specifically for performing PLS-SEM, a method used to analyze complex interactions between latent constructs and observable variables [74]. The user-centric design is praised for improving the data analysis and theoretical model validation processes in several domains, such as marketing, management, and social sciences. The platform is adaptable, capable of supporting formative and reflective measurement models to assess the reliability and validity of constructs. It also has advanced features for doing in-depth analyses, including mediation, moderation, and multi-group comparison [78].

Partial Least Squares Structural Equation Modeling (PLS-SEM) is a statistical method used to analyze the relationships between variables observed in the structural equation model in our research. It is particularly useful when dealing with complex models with many variables and relatively small sample sizes [74]. PLS estimates latent variables by finding linear combinations of observed variables (indicators) that explain the maximum variance in latent variables [77].

## 4 Results and discussion

### 4.1 Results

The measurement model in SEM plays a crucial role in validating the reliability and accuracy of the measures utilized in the study, ensuring they effectively represent the intended constructs [79]. This research employs a confirmatory factor analysis (CFA) for all the measures to evaluate the measurement model's convergent and discriminant validity and composite reliability. Convergent validity verifies that the indicators of a construct genuinely measure the same concept. In contrast, discriminant validity confirms that the constructs are distinct and separate from one another. Composite reliability, on the other hand, measures the consistency of the indicators.

This process is critical for confirming the data's integrity and the construct's validity before analyzing the structural relationships within the model [79]. To evaluate the measurement model's validity, this study scrutinized outer loadings, composite reliability (CR), average variance extracted (AVE), Cronbach's alpha, and Rho-A, following the guidelines by Hair et al. [75]. The results showed that the outer loadings for all measurement items were above the recommended threshold of 0.5. Additionally, the AVE for each latent variable exceeded the 0.5 benchmark. CR, Rho-A, and Cronbach's alpha values were all above 0.7, indicating strong convergent validity within the study's measurement model.

We use the Fornell-Larcker criterion that was introduced in 1981 for assessing the discriminant validity. This validity pertains to the extent to which a construct is differentiated from other constructs in the research. Discriminant validity is established when the square root of the average variance extracted (AVE) for each construct is greater than its correlation with other constructs in the model, as per the criterion. This suggests that a construct is more closely related to its own indicators than to the indicators of other constructs, confirming the distinct representation of each construct by its indicators. The Fornell-Larcker criterion is crucial for assessing the measurement model's adequacy in PLS-SEM. SmartPLS provides the necessary statistical tools to conduct these evaluations, aiding researchers in validating theoretical constructs effectively.

As indicated in Table 3, the bolded average square roots of the AVEs exhibit values that exceed the correlations between different constructs, with the exception of the GIC. Specifically, for GIC, the square root of its AVE stands at 0.721, while its correlations with EP, learning, and work-related ESM usage are noted at 0.807, 0.728, and 0.729, respectively. Consequently, the hetero-trait mono-trait (HTMT) ratio is employed to further confirm discriminant validity. The HTMT ratio should be less than 1.0 for acceptable discriminant validity. The HTMT values, italicized above the square roots of the AVEs, meet this criterion by being less than 1.0. These findings collectively affirm the discriminant validity of the measurement model.

**Table 3:** Inter-construct correlations and discriminant validity.

Constructs	Green intellectual capital	Hotel environmental performance	Learning	Vitality	Work-related ESM usage
Green intellectual capital	**0.721**	*0.866*	*0.771*	*0.777*	*0.779*
Hotel environmental performance	0.807	**0.812**	*0.810*	*0.814*	*0.775*
Learning	0.728	0.750	**0.873**	*0.936*	*0.773*
Vitality	0.716	0.734	0.846	**0.862**	*0.756*
Work-related ESM usage	0.729	0.715	0.718	0.686	**0.872**

Note. The bold font represents the square root of AVE values. The italics font represents the Hetero-Trait Mono-Trait values.

The study's interrelationships were examined via SEM. Specifically, the Beta-coefficient (*β*), as well as, the t and *p* values were reported to determine the significance of the relationships among constructs. Table 4 illustrates the findings of the path results. GIC had a significant positive impact on environmental performance (*β* = 0.084, t = 6.159, p = 0.00), learning (*β* = 0.075, t = 5.788, p = 0.00), and vitality (*β* = 0.074, t = 6.215, p = 0.00). Therefore, hypotheses H1a, H1b, and H1c were supported. Furthermore, vitality (*β* = 0.064, t = 2.653, p = 0.008) and learning (*β* = 0.078, t = 2.916, p = 0.004) significantly influence EP. Hence, H2a and H2b were supported. Moreover, Table 4 highlights the mediating role of learning (*β* = 0.046, t = 3.027, p = 0.003) and vitality (*β* = 0.046, t = 3.526, p = 0.00) in the relationship between GIC and hotel EP. Hence, H3a and H3b were supported. Over and above, the moderating role of work-related ESM usage in the relationship between GIC and learning and vitality was also assessed. The findings showed that work-related ESM usage had a significant positive impact on learning (*β* = 0.067, t = 5.983, p = 0.00) and vitality (*β* = 0.067, t = 5.277, p = 0.00). Furthermore, the impact of the interaction between GIC and work-related ESM usage on learning (*β* = 0.409, t = 5.983, p = 0.00) and vitality (*β* = 0.350, t = 5.277, p = 0.00) was significant. The findings indicated that work-related ESM usage moderates the relationship between GIC and TAW. These results revealed that the effect of GIC on learning and vitality is conditional on work-related ESM usage. Hence, H4a and H4b are supported.

**Table 4:** Hypotheses testing.

	SRMR = 0.042			
Path	*β*	t-value	p- value	Decision
Mediating model				
Direct effects				
H1a: Green intellectual capital →hotel environmental performance	0.084	6.158	0.00	Supported
H1b: Green intellectual capital →learning	0.075	5.788	0.00	Supported
H1c: Green intellectual capital → vitality	0.074	6.215	0.00	Supported
H2a: Vitality → hotel environmental performance	0.064	2.653	0.008	Supported
H2b: Learning → hotel environmental performance	0.078	2.916	0.004	Supported
Indirect effects				
H3a: Green intellectual capital→ vitality →hotel environmental performanc	0.067	5.277	0.00	Supported
H3b: Green intellectual capital→ learning →hotel environmental performance	0.067	5.983	0.00	Supported
Moderating model				
H4a: Interaction → learning	0.409	5.983	0.00	Supported
H4b: Interaction → vitality	0.350	5.277	0.00	Supported

Note. β: Unstandardized coefficients reported, interaction: green intellectual capital ∗work-related ESM usage.

Regarding the model fit, the root mean square residual (SRMR) is equal to 0.042, below the threshold of 0.08, ensuring adequate model fit [75].

This paper, which is underpinned by the JD-R theory and SEM, examined a moderated-mediation conceptual framework assessing the interrelationships among employees’ perceptions regarding GIC, EP, work-related ESM usage, and TAW by data being gathered from frontline workers working in Turkish hotels. The findings delineate strong support for all hypothesized relationships.

## 5 Discussion

The research findings show that employees' perceptions of their hotel's GIC have a considerable impact on their TAW, which then positively affects their perceptions of the hotel's EP. Specifically, the relationship between employee perceptions of GIC and EP aligns with findings from Malik et al. [11] and Nisar et al. [1], supporting the notion that JR, plays a crucial role in promoting employee vitality, learning, and personal growth. This leads to TAW and better sustainable practices [50; 51]. It's proposed that this perception can foster a positive workplace atmosphere, encouraging employee vitality, learning, and growth [4], which in turn bolsters work-related performance [58].

Secondly, the research indicates that employees' perceptions of the hotels' EP directly stem from their sense of vitality and the opportunities for learning at work. These insights align with Karatepe et al. [4] and Ozturk et al. [35], where the JD-R theory elucidates that a motivated and energized workforce enhances the organization's environmental outcomes as a result of their TAW. Furthermore, the SEM supports that employees who are both energized and equipped with learning and growth opportunities are likely to thrive and, consequently, positively impact sustainable practices [50; 51] and harbor favorable views on their organizations' environmental efforts [4].

Thirdly, the research demonstrates that TAW acts as a mediating factor between employees' perceptions of hotels' GIC and their EP. Specifically, employees who possess the skills, knowledge, and capabilities related to EI are likely to influence their perception of the hotel's environmental efforts [1] and experience TAW, facilitating self-development and valuable outcomes [57]. The JD-R theory posits that GIC, encompassing human, structural, and relational capital, serves as a JR impacting outcomes like EP [4; 77]. Concurrently, according to the SEM, job-related resources and contextual factors are predictive of TAW [36; 37], thereby boosting sustainable outcomes and job performance [47; 50]. This suggests that employees' perceptions of GIC inspire a sense of vitality, learning, and growth [4], encouraging positive views on hotels' environmental practices [23 35].

Lastly, the research uncovers the moderating role of employees' perceptions of work-related ESM usage on the dynamic between their views on hotels' GIC and TAW. Notably, work-related ESM facilitates the exchange of information, knowledge, and skills [26], enhances employee performance and productivity [63], and fosters TAW [18; 62]. Thus, work-related ESM usage aids in the dissemination of skills, knowledge, and information regarding EI, promoting TAW [18; 61] and positively affecting employees' perceptions of organizational EP [4].

## 6 Conclusion

This research fills a critical void in existing studies within the hospitality industry by exploring the relatively untouched area of how employees view hotels' GIC and its various possible impacts. Anchored in the theory of TAW, this study presents a conceptual framework that suggests the mediating of TAW between employees' views on hotels' GIC and their EP, alongside the moderating effect of the use of work-related ESM. The analysis of data gathered from frontline staff in Turkish four- and five-star hotels through SEM yielded significant results. It was discovered that employees' views on hotels' GIC positively affected their TAW, which in turn, improved their views on the hotels' EP. Moreover, TAW was identified as a crucial mediator in the relationship mentioned earlier. The research also highlighted the moderating influence of work-related enterprise social media usage on the relationship between employees' perceptions of hotels' GIC and their TAW. These outcomes offer important insights into the complex interactions between GIC, employee thriving, and EP in the hospitality industry.

### 6.1 Theoretical implications

Our paper contributes to current knowledge in several ways. First, the current paper fills in the gaps in the literature by revealing the positive effect of employees' perceptions regarding hotels' GIC on their perceptions of EP and TAW. These findings are crucial since the existing literature suggests that GIC is a new concept that needs more research to understand its role in promoting sustainable development [1; 11]. Accordingly, our study promotes an understanding of the consequences of employees' perceptions regarding GIC in the hospitality context. In particular, our study indicated that employees' perceptions regarding hotels' GIC would influence their thriving and might enhance their perceptions of hotels' EP. Therefore, employees’ perceptions regarding GIC would help them fulfill their tasks in an environmentally friendly manner.

Second, as reported above, TAW was utilized as a mediator, linking employees' perceptions regarding hotels' GIC and EP. This study fills in an important gap since previous studies had gauged the relationship between GIC and EP, utilizing EB as a mediator [1] or moderator [80]. Accordingly, the study's results contribute to the literature since no empirical studies have linked employees' perceptions of GIC to EP via the mediating role of TAW. Therefore, assessing this mediating relationship in our study represents a novelty.

Third, in today's competitive environment, organizations tend to rely on ESM to share knowledge, skills, and information among employees [15; 26]. Accordingly, our paper has utilized work-related ESM usage as a moderator of the effect of employees' perceptions regarding hotels' GIC on TAW. Therefore, our paper fills in an important gap because there was no previous study that utilized work-related ESM usage as an intermediary GIC and TAW in the hospitality context.

### 6.2 Managerial implications

The results provide multiple managerial implications for managers and policymakers. First, the results revealed that employees' perceptions of GIC are a strong predictor of employees' perceptions regarding EP and TAW. Moreover, the findings indicated that TAW significantly influences employees' perceptions of hotels' EP. Accordingly, HR managers should tailor training programs to enhance employees' perceptions regarding GIC. This would help the top management create a workforce aligned with the organization's green objectives. Moreover, managers should design, develop, and implement innovative green strategies to train and equip workers with the needed skills through conducting formal and informal educational programs, providing incentives for eco-friendly behaviors, and building green relationships with other business partners. In particular, while establishing strategies and operational practices, top management has to prioritize environmental issues and diffuse them through the organization's ESM, which would motivate, energize, and enhance employees' personal growth and promote their perceptions of the organization's EP. Consequently, managers could communicate their commitment to the environment to their employees, thus providing them with the needed resources to enhance their perceptions regarding hotels' GIC, vitality, and learning. Furthermore, the top management should encourage workers to present green ideas and participate in green behaviors and initiatives. Through capitalizing on the effective usage of ESM, managers should promote collaboration among different departments and motivate employees to establish cross-functional teams to work in eco-friendly programs and sustainable projects. Over and above, the top management would utilize the ESM to inform employees and all stakeholders about the hotel's GIC investments and environmental contributions. Moreover, the top management should collect feedback from employees regularly to identify areas of improvement while they are assessing the effectiveness of their investments in GIC.

In addition, top management should create internal structural capital through building a green organizational culture and information systems. By building an environmentally aware culture within companies, employees are more likely to feel increased passion, motivation, and a drive to acquire the information and skills needed to fulfill sustainable objectives.

### 6.3 Limitations and future recommendations

Future investigations should aim to overcome the constraints identified in our current study. Initially, the research examined the interrelationships among employees' perceptions of GIC, TAW, EP, and work-related ESM usage. However, causality could not be explored since this paper was a cross-sectional study. As a remedy, future longitudinal studies might be conducted. Second, the study's constructs were assessed and rated by frontline employees, and that might lead to response bias or conformity bias. Therefore, future research could investigate our conceptual model from supervisors' and managers' perspectives. Third, our paper has treated TAW as a mediator between employees' perceptions regarding GIC and hotels' EP and investigated the moderating role of work-related ESM usage. Accordingly, future research would examine the mediating role of pro-environmental technologies, organizational infrastructure, and green knowledge management that would contribute to organizations' EP. Fourth, researchers claimed that GIC is a novel construct that needs further investigation [1]. Accordingly, future research would investigate the antecedents of GIC that would help managers expand their employees' expertise, knowledge, and skills, thus boosting green output. Finally, the paper's conceptual model is examined via data collected from frontline employees working in Turkish hotels. Therefore, future studies could replicate the model in other service contexts to verify its reliability and adaptability.

## CRediT authorship contribution statement

**Ertac Gulakdeniz:** Writing – original draft. **Georgiana Karadas:** Project administration.

## Disclosure statement

No potential conflict of interest was reported by the author(s).

## Data availability statement

Data will be made available on request.

## Declaration of competing interest

The authors declare that they have no known competing financial interests or personal relationships that could have appeared to influence the work reported in this paper.
